# D-serine increases adult hippocampal neurogenesis

**DOI:** 10.3389/fnins.2013.00155

**Published:** 2013-08-29

**Authors:** Sebastien Sultan, Elias G. Gebara, Kristell Moullec, Nicolas Toni

**Affiliations:** Department of Fundamental Neurosciences, University of LausanneLausanne, Switzerland

**Keywords:** adult neurogenesis, dentate gyrus, d-serine, stem cell niche, stem cell factor, astrocytes

## Abstract

Adult hippocampal neurogenesis results in the continuous formation of new neurons and is a process of brain plasticity involved in learning and memory. The neurogenic niche regulates the stem cell proliferation and the differentiation and survival of new neurons and a major contributor to the neurogenic niche are astrocytes. Among the molecules secreted by astrocytes, D-serine is an important gliotransmitter and is a co-agonist of the glutamate, N-methyl-D-aspartate (NMDA) receptor. D-serine has been shown to enhance the proliferation of neural stem cells *in vitro*, but its effect on adult neurogenesis *in vivo* is unknown. Here, we tested the effect of exogenous administration of D-serine on adult neurogenesis in the mouse dentate gyrus. We found that 1 week of treatment with D-serine increased cell proliferation *in vivo* and *in vitro* and increased the density of neural stem cells and transit amplifying progenitors. Furthermore, D-serine increased the survival of newborn neurons. Together, these results indicate that D-serine treatment resulted in the improvement of several steps of adult neurogenesis *in vivo*.

## Introduction

Adult mammalian neurogenesis results in the formation of new neurons principally in the olfactory bulb and the dentate gyrus (DG) of the hippocampus (Altman, 1969). The process of hippocampal neurogenesis consists in several steps: (1) Cell proliferation: Neural stem cells reside in the subgranular zone (SGZ) of the DG, have a radial glia-like (RGL) morphology and express the glial fibrillary acidic protein (GFAP) and nestin. They give rise to GFAP-negative, nestin-positive, Tbr2-positive transit-amplifying progenitors (TAP) (Gage, 2000; Yao et al., 2012). These highly proliferative TAPs then give rise to neuroblasts. (2) The differentiation of the newly formed cells into neuronal lineage by the expression of the immature neuronal marker doublecortin (DCX) followed by the mature neuronal marker Neu-N, the cell polarization and extension of dendrites and axons (Gage, 2000; Van Praag et al., 2002; Laplagne et al., 2006; Yao et al., 2012). (3) The migration of the new neurons into the granule cell layer. (4) The activity-dependent survival of the newly formed neurons (Tashiro et al., 2006). (5) Their functional integration into the hippocampal network (Toni et al., 2007, 2008; Toni and Sultan, 2011) and participation to mechanisms of learning and memory (Aimone et al., 2010).

Each of these steps is highly regulated by signals within the stem cells' specialized environment, called the neurogenic niche. The niche is constituted by several cell types, including astrocytes, the stem cell's progenies, oligodendrocytes, endothelial cells, microglia, mature and immature neurons (Shihabuddin et al., 2000; Song et al., 2002; Zhao et al., 2008; Bonaguidi et al., 2011). All together these cells release specific factors, like Transforming Growth Factor-β (TGF-β) (Kreutzberg, 1996a,b), basic Fibroblast Growth Factor (bFGF) (Wagner et al., 1999), Vascular Endothelial Growth Factor (VEGF) and also Brain Derived Neurotrophic Factor (BDNF) (Bergami et al., 2008; Lee and Son, 2009), which are all involved in the regulation of the niche homeostasis. Of particular interest, astrocytes seem to play a special role in the regulation of the neurogenic niche, by expressing membrane-associated or secreted pro-neurogenic factors involved in the proliferation or neuronal differentiation of adult neural stem cells (Song et al., 2002; Lie et al., 2005; Platel et al., 2010; Ashton et al., 2012).

A particularly interesting factor secreted by astrocytes, D-serine is abundant in the hippocampus and especially in hippocampal astrocytes (Schell et al., 1995) where it is stored in vesicles (Bergersen et al., 2012; Martineau et al., 2013). D-serine is a co-agonist of the NMDA receptor (Mothet et al., 2000) and its calcium-dependent release participates to the expression of long-term potentiation (LTP) in the hippocampus (Yang et al., 2003; Henneberger et al., 2010). D-serine crosses the blood-brain barrier, since the exogenous administration of D-serine increases extracellular and intracellular brain D-serine levels (Pernot et al., 2012). Interestingly, D-serine administration enables LTP (Bashir et al., 1990; Oliver et al., 1990; Watanabe et al., 1992; Duffy et al., 2008) and can reverse the aging-related deficits in LTP expression and learning performances (Mothet et al., 2006; Devito et al., 2011), suggesting that D-serine could be targeted as a cognitive enhancer (Collingridge et al., 2013).

Here, we investigated the effect of exogenous D-serine administration on adult neurogenesis. We found that administration for 8 days increased cell proliferation, the number of RGL cells and the number of TAPs in the dentate gyrus of adult mice. Similarly, *in vitro*, D-serine increased the number of stem/progenitor cells. Finally, when administered during the fourth week after cell division, D-serine increased the survival of new neurons.

## Experimental procedures

### Animals and D-serine administration

All experimental protocols were approved by the Swiss animal experimentation authorities (Service de la consommation et des affaires vétérinaires, Chemin des Boveresses 155, 1066; Epalinges, Switzerland, permit number: 2301). Every effort was made to minimize the number of animals used and their suffering. Animals used for the study were adult male of 8 weeks of age at the beginning of the experiment. All animals were housed in a 12 h light/12 h dark cycle with free access to food and water and controlled temperature (22°C) conditions. C57Bl/6j mice were purchased from Janvier (le Genest Saint Isle, France), nestin-GFP mice were a kind gift from the laboratory of K. Mori (PRESTO, Kyoto, Japan) (Yamaguchi et al., 2000). These mice express the green fluorescent protein (GFP) under the stem cell-specific promoter nestin. GFAP-GFP mice were a kind gift from the laboratory of Helmut Kettenmann (Max-Delbruck center, Berlin, Germany) (Nolte et al., 2001). They express GFP under the control of the astrocyte-specific promoter GFAP. D-serine was prepared fresh every day and diluted in water containing 0.9% NaCl. Mice were injected intraperitoneally every day with 50 mg/kg of D-serine (Sigma-Aldrich) for 8 consecutive days or with vehicle (0.9% NaCl in water).

### BrdU administration and immunohistochemistry

Mice were injected intraperitoneally with 5-bromo-2-deoxyuridine (BrdU, Sigma-Aldrich, Buchs, Switzerland) at a concentration of 100 mg/kg in saline, 3 times at 2-h intervals. Two hours after the last injection, mice were sacrificed to examine cell proliferation (Mandyam et al., 2007; Taupin, 2007; Yang et al., 2011; Gao and Chen, 2013; Sultan et al., 2013). Briefly, mice were injected intraperitoneally with a lethal dose of pentobarbital (10 mL/kg, Sigma, Switzerland) and then perfused with 50 ml of 0.9% saline solution followed by 100 mL of 4% paraformaldehyde (Sigma-Aldrich, USA) dissolved in 0.1 M Phosphate Saline Buffer (PBS, pH 7.4). Their brains were dissected, postfixed overnight at 4°C, cryoprotected 24 h in 30% sucrose solution (Sigma-Aldrich, USA) and rapidly frozen. Then coronal sections were performed at a thickness of 40 μm with a microtome-cryostat (Leica MC 3050S) and slices were stored in cryoprotectant (30% ethylene glycol and 25% glycerin in PBS 0.1 M) at −20°C until processing for immunostaining as previously described (Thuret et al., 2009). Immunochemistry was performed on every 6th sections of the dentate gyrus. Briefly, sections were washed 3 times in PBS 0.1 M and blocking of non-specific binding was achieved by incubating in PBS 0.1 M containing 0.3% Triton-X 100 and 10% normal serum. BrdU immunohistochemistry was preceded by DNA denaturation by incubation in formic acid 50% formamide/ 50% 2X SSC buffer (2X SSC is 0.3 M NaCl and 0.03 M sodium citrate, pH 7.0) at 65°C for 2 h, rinsed twice in 2X SSC buffer, incubated in 2 M HCl for 30 min at 37°C, and rinsed in 0.1 M borate buffer pH 8.5 for 10 min. Then, sections were incubated at 4°C with one of the following primary antibodies: mouse monoclonal anti-BrdU (48 h, 1:250, Chemicon International, Dietikon, Switzerland), goat anti-DCX (1:500, Santa Cruz biotechnology, sc-8066), rabbit anti-Ki-67 (48 h, 1:200, Abcam, ab15580), rabbit anti-Tbr2 (1:200, Abcam, ab23345), rabbit anti-GFAP (1:500, Invitrogen, 180063) mouse anti-Neu-N (Chemicon international 1:1000). Sections were then incubated for 2 h at room temperature with the following fluorescent secondary antibody: goat anti-mouse Alexa-594 (1:250, Invitrogen), goat anti-rabbit 594 (1:250, Invitrogen), donkey anti-goat Alexa-555 (1:250 Invitrogen). After immunostaining, one minute incubation of slices into 4,6 diamidino-2-phenylindole (DAPI) was used to reveal nuclei.

### Image analysis

Images were collected with a Zeiss confocal microscope (Zeiss LSM 710 Quasar Carl Zeiss, Oberkochen, Germany) and cell counts were performed using stereology, as previously described (Thuret et al., 2009). Briefly, for each animal, a 1-in-6 series of section between −1.3 and −2.9 mm from the Bregma was stained with the nucleus marker DAPI and used to measure the volume of the granule cell layer. The granule cell area was traced using Axiovision (Zeiss, Germany) software and the granule cell reference volume was determined by multiplying the area of the granule cell layer by the distance between the sections sampled (240 μm). All cells were counted in the entire thickness of the sections in a 1-in-6 series of section (240 μm apart) with a 40× objective. All cells were counted blind with regard to the mouse status. The number of immunolabeled cells was then related to granule cell layer sectional volume and multiplied by the reference volume to estimate the total number of immunolabeled cells. Cells expressing BrdU, Ki-67, DCX or Tbr2 were counted in the granule cell layer, whereas cells expressing GFAP (Figures 3C,D) were counted in the whole dentate gyrus. BrdU colocalization with the neuronal marker Neu-N was analyzed by confocal microscopy and was confirmed on single optical sections, for 50–60 cells per animal. The proportion of double-labeled cells was then obtained for each animal and then averaged for each group.

### Cell culture

Astroyctes primary culture: Cerebral cortices from 3 postnatal day 0 (P0) rats were mechanically dissociated for homogenization, cells were pooled and seeded onto 75 cm^2^ flasks in Dulbecco's Modified Eagle Medium (DMEM) glutamax (Invitrogen, USA), 15% foetal bovine serum with penicillin/streptomycin (Invitrogen, USA). Cells were grown for 10–12 days in a humidified 5% CO_2_ incubator at 37°C. At confluence, flasks were shaken at 250 rpm on an orbital shaker for 2 h to remove microglia. Adult neural progenitor cells (NPCs) expressing the red fluorescent protein (RFP) are a kind gift from the laboratory of Fred Gage (Salk Institute, San Diego, USA). They were originally isolated from the dentate gyrus of adult Fisher 344 rats and cultured as previously described (Ray and Gage, 2006). RFP expressing NPCs were plated on coated 12 mm coverslips in a 24-well culture plate, at a density of 2,000,000 cells/mL (80 μL per well). Medium was changed daily and supplemented with 2 μ M FGF2. Three wells per condition were used. In a first experiment, the effect of D-serine was tested on NPCs and then, in a second, independent experiment, on NPCs-astrocytes co-cultures. Twenty-four hours after plating, the medium was replaced by fresh culture medium, and then treated daily with D-serine (50 μM, diluted in culture media) during 8 days. Control cultures were treated with the same volume of vehicle. After this, cells were fixed with 4% paraformaldehyde for 20 min, washed and the coverslips were immunostained and mounted.

### *In vitro* cell quantification

Images were acquired using confocal microscopy. The number of RFP+ and GFAP+ cells was counted in 4 selected fields, systematically placed in the same positions relative to the coverslips' edges. The total number of cells was divided by the total area of the selected fields to obtain an average cell density per well that was then multiplied by the total surface area of the coverslip to obtain an estimate of the total number of cells per coverslip. This number of cells was then compared to the number of cells that were plated in the wells to obtain a percentage of increase in cell number (Gebara et al., 2013).

### Statistical analysis

Hypothesis testing was two-tailed. All analyses were performed using JMP10 software. First, Shapiro-Wilk tests were performed on each group of data to test for distribution normality. The distribution was normal for all data. The analysis was performed using parametric tests (One-Way ANOVA followed by a *post hoc* bilateral Student's *t*-test). For two-sample comparisons, the equality of variances of the groups was tested and the adequate unpaired *t*-test was used. Data is presented as mean ± SEM.

## Results

### D-serine increased cell proliferation in the dentate gyrus

**Figure 1:**
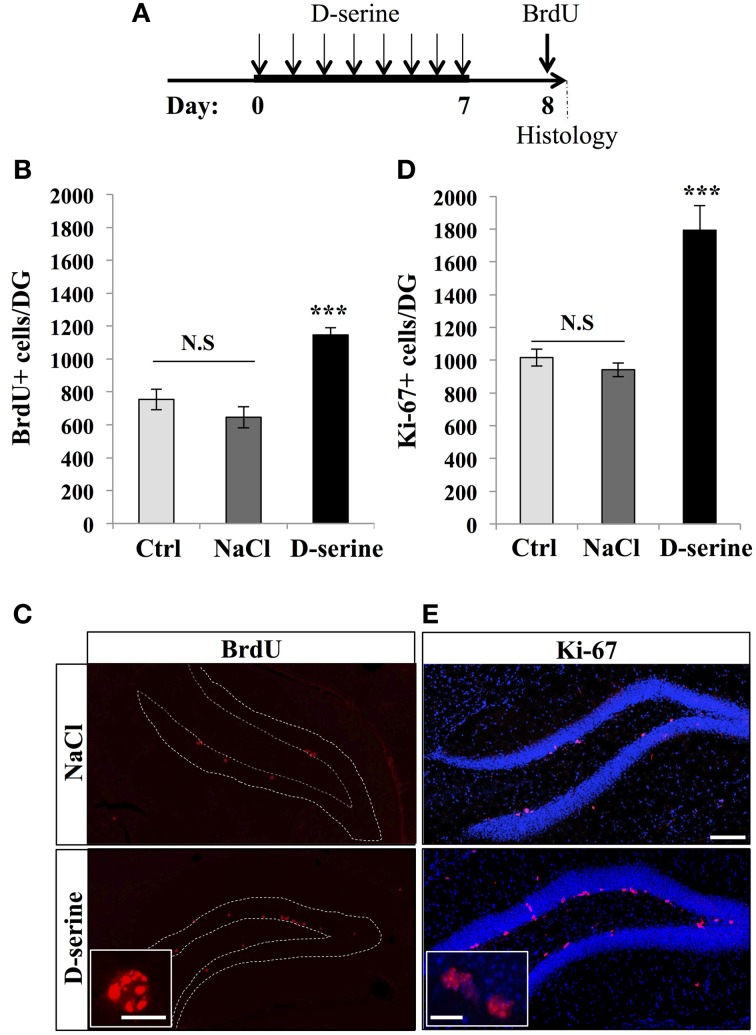
**D-serine increased cell proliferation in the dentate gyrus**. **(A)** Experimental timeline: Mice were injected intraperitoneally every day with 50 mg/kg of D-serine, for 8 consecutive days. One day after the last injection, mice were pulsed with BrdU (100 mg/kg, 3 injections every 2 h) and, 2 h later, euthanized and prepared for immunohistochemistry. **(B)** Histogram showing the number of BrdU-expressing cells per dentate gyrus of animals injected with D-serine, NaCl or not injected (Ctrl). Animals: *n* = 4 per group. **(C)** Confocal maximal projection micrographs of hippocampal sections immunostained for BrdU in the two injected groups (D-serine and NaCl). Inset: Higher magnification micrograph of a BrdU-expressing cell. **(D)** Histogram showing the number of Ki-67–expressing cells. Animals: *n* = 4 per group. **(E)** Confocal maximal projection micrographs of hippocampal sections immunostained for Ki-67 in the two experimental groups (D-serine and NaCl). Inset: Higher magnification micrograph of Ki-67-expressing cells. Blue: Dapi staining. Each value represents the mean ± SEM; *post-hoc* bilateral Student's *t*-test, (^***^p < 0.001), N.S., non-significant (*p* > 0.05). Scale bar: 100 μm, insets 10 μm.

We first examined the effect of D-serine on cell proliferation in the dentate gyrus of adult C57Bl/6 mice. Eight-week-old mice were injected with one daily intraperitonal injection of D-serine (50 mg/kg), for 8 days, as this regimen is known to increase extracellular D-serine brain levels (Fukushima et al., 2004; Ferraris et al., 2008) and improve learning performances in mice (Bado et al., 2011; Filali and Lalonde, 2013) (Figure 1A). Control mice were injected with the same volume of the vehicle (0.9% NaCl), or received no injection. One day after the last injection, all mice received 3 intraperitoneal injections of the cell proliferation tracer BrdU (100 mg/kg) at 2-h intervals and were sacrificed 2 h after the last BrdU injection. Brains were sectioned and immunostained for BrdU and the proliferation marker Ki-67 and the number of immunoreactive cells was counted in the granule cell layer of the dentate gyrus. D-serine injections significantly increased the number of BrdU-expressing cells [Figures 1B,C, One-Way ANOVA, *F*_(2, 11)_ = 20.64, *p* < 0.001; *post-hoc* bilateral Student's *t*-test between groups *p* < 0.001] and the number of Ki-67-expressing cells [Figures 1D,E, One-Way ANOVA, *F*_(2, 11)_ = 24.90, *p* < 0.001; *post-hoc* bilateral Student's *t*-test between groups, *p* < 0.001]. To test whether the D-serine-induced increase in cell proliferation was specific to the dentate gyrus, we counted the number of Ki-67-expressing cells in the S1 area of the somatosensory cortex. D-serine treatment did not change the density of Ki-67+ cells in the somatosensory cortex (NaCl: 2.54 ± 0.14 × 10^−6^ cells/μ m^3^ vs. D-serine: 2.83 ± 0.28 × 10^−6^ cells/μ m^3^, Student's *t*-test *p* = 0.3). No difference was found between non-injected and NaCl-injected animals (*post-hoc* bilateral Student's *t*-test *p* = 0.21 for BrdU+ cells and *p* = 0.58 for Ki-67+ cells). To test whether the increased number of BrdU- or Ki-67-expressing cells could be caused by a change in hippocampal volume upon D-serine treatment, we measured the volume of the granule cell layer of all mice. We did not detect any difference between treated and control animals [One-Way ANOVA, *F*_(2, 11)_ = 2.20, *p* = 0.166, control animals: 0.17 ± 0.006 mm^3^, animals injected with NaCl 0.18 ± 0.04 mm^3^, injected with D-serine 0.17 ± 0.03 mm^3^ respectively, *n* = 4 animals per group], indicating that the increased numbers of BrdU and Ki-67 cells reflected an increase in cell proliferation.

**Figure 2:**
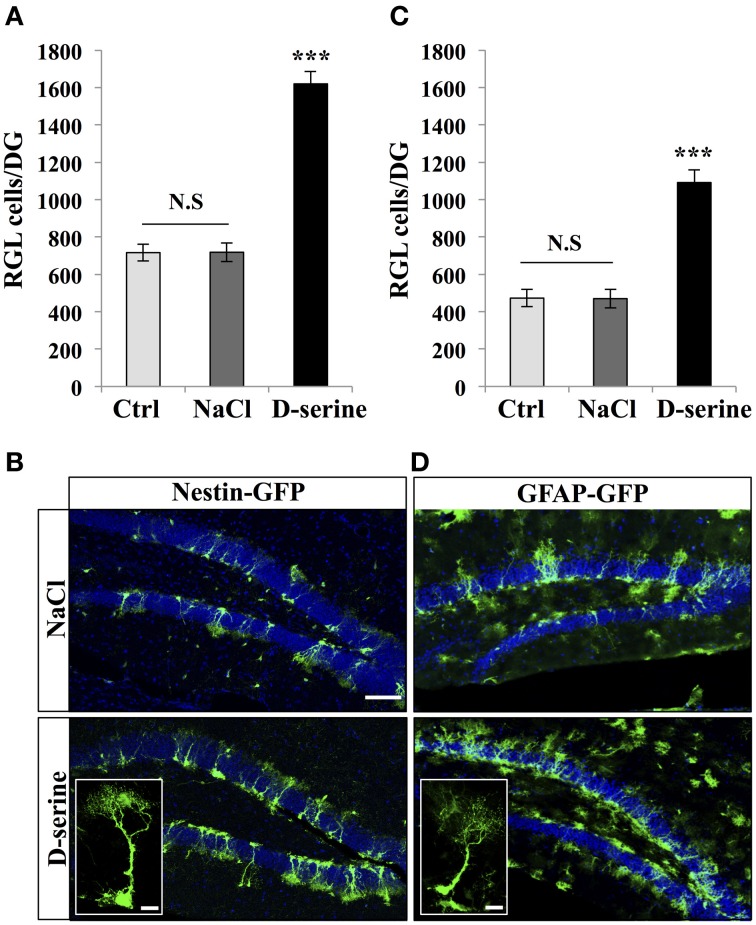
**D-serine increased the number of RGL cells**. **(A)** Histogram showing the number of RGL cells per dentate gyrus of nestin-GFP mice injected with D-serine, NaCl or not injected (Ctrl). Animals *n* = 4 per group. **(B)** Confocal maximal projection micrographs of hippocampal sections in the two injected groups (D-serine and NaCl). Inset: higher magnification confocal micrograph of a RGL cell. **(C)** Histograms showing the number of RGL cells in the dentate gyrus of GFAP-GFP mice injected with D-serine, NaCl and in control, non-injected animals (Ctrl). Animals: *n* = 4 animals per groups. **(D)** Confocal maximal projection micrographs of hippocampal sections from the two experimental groups. Inset: higher magnification micrograph of a RGL cell. Blue: Dapi staining. Each value represents the mean ± SEM; *post-hoc* bilateral Student's *t*-test, (^***^p < 0.001), N.S., non-significant (*p* > 0.05). Scale bar: 100 μm. Insets 10 μm.

**Figure 3:**
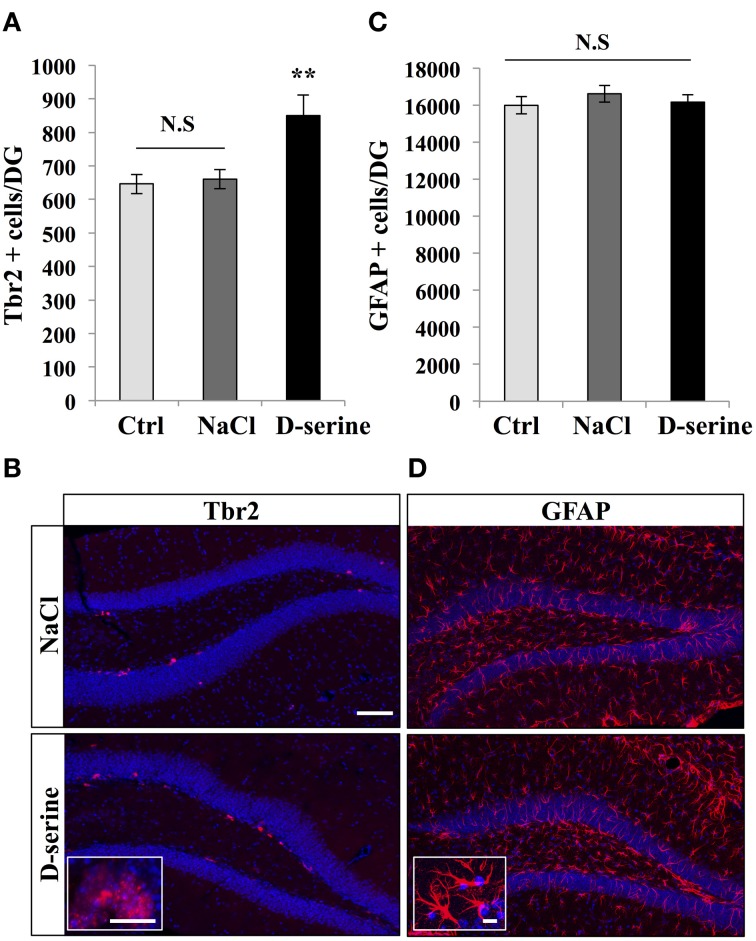
**D-serine increased the number of Tbr-2-expressing cells**. **(A)** Histogram showing the number of Tbr2-expressing cells in the granule cell layer of the dentate gyrus of mice injected with D-serine or NaCl and in control, non-injected animals (Ctrl). Animals: *n* = 4 animals per group. **(B)** Confocal maximal projection micrographs of hippocampal sections immunostained for Tbr2 from the two injected groups. Inset: higher magnification micrograph of Tbr-2-immunolabeled cells. **(C)** Histogram showing the number of GFAP-expressing cells per dentate gyrus of injected mice (D-serine and NaCl) and in control, non-injected animals (Ctrl). Animals: *n* = 4 per groups. **(D)** Confocal maximal projection micrographs of hippocampal sections immunostained for GFAP from the two experimental groups. Inset: higher magnification micrograph of a GFAP-immunostained cell. Blue: Dapi staining. Each value represents the mean ± SEM; *post-hoc* bilateral Student's *t*-test, (^**^p < 0.01), N.S., Non-significant (*p* > 0.05). Scale bar: 100 μm, insets 10 μm.

We then examined the effect of D-serine on the main proliferative cells in the SGZ: the type-1 radial glia-like (RGL) stem cells and TAPs (TAPs; Figures 2, 3). To identify RGL cells, we used a transgenic mouse expressing GFP under the stem cell-specific promoter nestin (Yamaguchi et al., 2000). GFP-expressing RGL cells of the dentate gyrus were readily identifiable by their morphology, consisting of a nucleus located in the subgranular zone, a large processes extending through the granule cell layer and branching into the proximal part of the molecular layer (Kriegstein and Alvarez-Buylla, 2009), (Figure 2B). With immunostaining, we confirmed that these cells expressed nestin, GFAP, and sox-2 (data not shown). D-serine treatment significantly increased the number of RGL cells in the dentate gyrus as compared to control or vehicle treatment [Figure 2A, One-Way ANOVA, *F*_(2, 11)_ = 90.36, *p* < 0.001; *post-hoc* bilateral Student's *t*-test between groups *p* < 0.001]. RGL cells were also identified in GFAP-GFP mice (Nolte et al., 2001; Figure 2D). Similarly, in GFAP-GFP mice, D-serine induced an increase in RGL cell number in the subgranular zone [Figures 2C,D, One-Way ANOVA, *F*_(2, 11)_ = 35.20, *p* < 0.001; *post-hoc* bilateral Student's *t*-test between groups *p* < 0.001]. Here too, we did not detect any difference in the volume of the granule cell layer between treated and control animals in both nestin-GFP and GFAP-GFP mice [nestin-GFP mice: One-Way ANOVA, *F*_(2, 11)_ = 0.34, *p* = 0.7 and GFAP-GFP mice One-Way ANOVA, *F*_(2, 11)_ = 1.6, *p* = 0.24, respectively *n* = 4 animals per group]. To examine whether the proliferation potential of the RGL cells was modified by treatment, we labeled GFAP-GFP mice with the proliferation marker Ki-67. We analyzed 4 mice per group and a total of 615 RGL cells for the D-serine group and 317 RGL cells for the NaCl group. D-serine treatment showed a significant increase of the percentage RGL cells that expressed Ki-67 as compared to NaCl treatment (Data not shown, D-serine treatment 5.54% ± 0.15 vs. NaCl 4.16% ± 0.43, Student's *t*-test *p* < 0.05).

We next examined the effect of D-serine on TAPs identified by immunohistochemistry against T-brain gene-2 (Hodge et al., 2008). D-serine significantly increased the number of Tbr2-expressing cells in the granule cell layer [Figures 3A,B, One-Way ANOVA, *F*_(2, 11)_ = 7.18, *p* = 0.013; *post-hoc* bilateral Student's *t*-test between groups *p* < 0.01]. The effect was specific to proliferating cells, since D-serine treatment did not change the number of GFAP-immunolabeled astrocytes of the whole dentate gyrus, i.e., including hilus, granule cell layer and molecular layer [Figures 3C,D, One-Way ANOVA, *F*_(2, 11)_ = 0.54, *p* = 0.59]. Together, these results indicate that D-serine increased cell proliferation in the SGZ and increased the number of RGL cells and TAPs.

### D-serine increased the proliferation of NPCs *in vitro*

**Figure 4:**
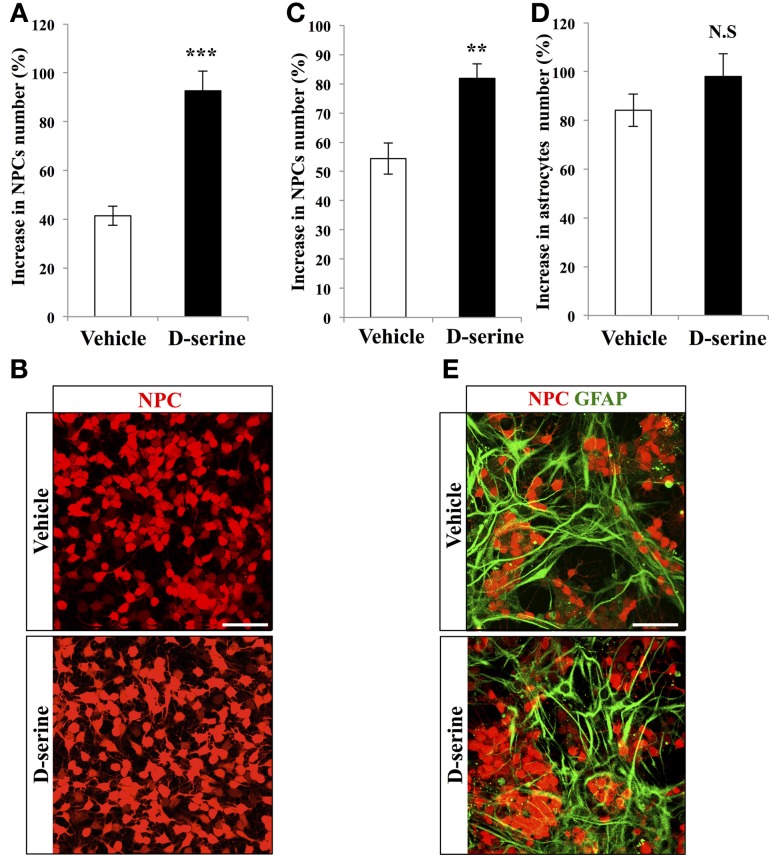
**D-serine increased the number of NPCs *in vitro***. **(A)** Histogram of the increase in NPCs number (% of the number of plated cells), upon treatment with D-serine or vehicle. **(B)** Confocal maximal projection micrographs of RFP-expressing NPCs for each condition. **(C,D)** Histograms showing the proportion of increase in NPCs **(C)** and in GFAP-expressing astrocytes **(D)** in co-cultures treated with D-serine or vehicle. **(E)** Confocal maximal projection micrographs of NPCs-Astrocytes co-cultures in the two conditions. Each value represents the mean ± SEM; *post-hoc* bilateral Student's *t*- test, (^***^p < 0.001, ^**^p < 0.01), N.S., non-significant (*p* > 0.05). Scale bars: 100 μm.

To test the effect of D-serine directly on progenitor cells, we performed *in vitro* experiments on purified NPCs. We plated the same number of RFP-expressing NPCs from the adult dentate gyrus in each well, treated them for 8 days with D-serine (50 μ M, daily) or vehicle (culture medium) and then counted the number of remaining NPCs, that we then expressed as the proportion of increase in cell number from the plated cell number. D-serine significantly increased the number of NPCs (Figures 4A,B, bilateral Student's *t*-test *p* < 0.001). On another set of experiments, we tested the effect of D-serine on NPCs in presence of astrocytes, by examining the effect of D-serine on co-cultures of astrocytes with NPCs. Similarly to purified cultures, co-cultures treated for 8 days with D-serine showed a greater number of NPCs (Figures 4C,E, bilateral Student's *t*-test *p* < 0.01). However, the number of GFAP-immunolabeled astrocytes remained unchanged as compared to control co-cultures (Figures 4D,E, bilateral Student's *t*-test *p* = 0.19). Thus, D-serine increased the number of NPCs, but not of astrocytes.

### D-serine increased the number of immature neurons and the survival of newborn neurons

**Figure 5:**
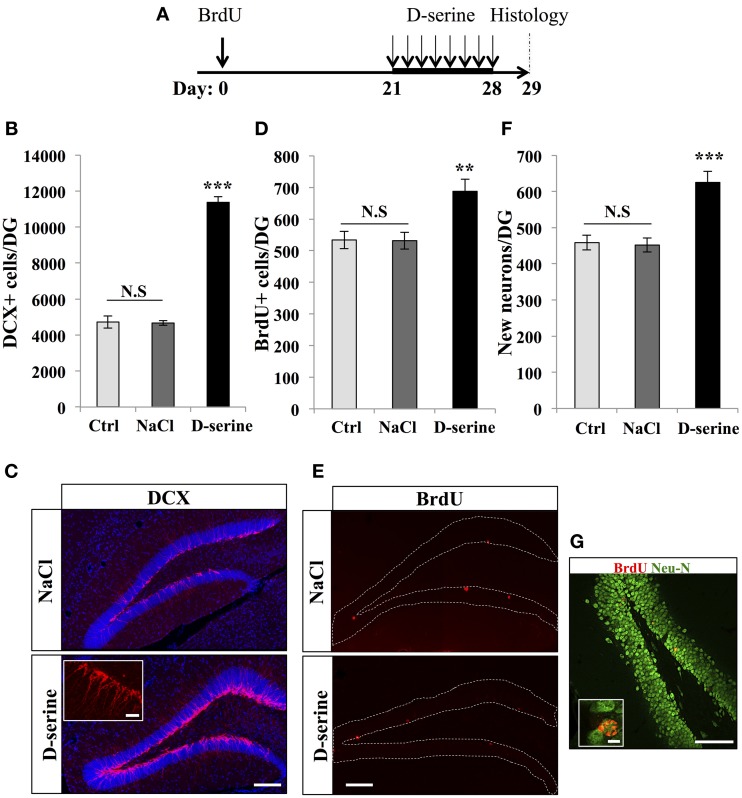
**D-serine increased the number of DCX-expressing cells and the survival of new neurons**. **(A)** Experimental timeline: Mice were injected with BrdU (100 mg/kg) 3 times at 2 h interval. Twenty-one days later, mice received one injection of D-serine (50 mg/kg) every day for 8 consecutive days. One day after the last injection, mice were euthanized and prepared for histology. **(B)** Histogram showing the number of DCX-immunolabeled cells in the dentate gyrus of mice injected with D-serine or NaCl and in control, non-injected animals (Ctrl). Animals: *n* = 4 per groups. **(C)** Confocal maximal projection micrographs of hippocampal sections immunostained for DCX from the two experimental groups. Inset: higher magnification micrograph of a DCX-immunolabeled group of cells. **(D)** Histogram of the number of BrdU-immunolabeled cells in the dentate gyrus of mice injected with D-serine or NaCl and non-injected animals (Ctrl). Animals: *n* = 4 per groups. **(E)** Confocal maximal projection micrographs of hippocampal sections immunostained for BrdU from the two experimental groups. **(F)** Histogram showing the number of new born neurons in the dentate gyrus of each group. Animals: *n* = 4 per group. **(G)** Single optical section confocal micrograph of BrdU- and Neu-N-immunolabeled cells in the dentate gyrus. Inset: higher magnification confocal micrograph of a BrdU-labeled cell expressing Neu-N. Blue: dapi staining. Each value represents the mean ± SEM; *post-hoc* bilateral Student's *t*-test (^**^p < 0.01, ^***^p < 0.001), N.S., non-significant (*p* > 0.05). Scale bars: 100 μm, insets 10 μm.

Next, to test whether D-serine affected neuronal differentiation and survival, we examined the number of immature neurons and the fate of the dividing cells, which matured under a D-serine treatment. Adult mice were injected with BrdU (intraperitoneal at 100 mg/kg, 3 injections at 2-h intervals) and were treated with D-serine from 22 to 29 days after the last BrdU injection. One day after the last injection, mice were sacrificed and brain sections were immunostained for the immature neuronal marker doublecortin (DCX), the mature neuronal marker Neu-N and BrdU (Figure 5). D-serine significantly increased the number of DCX-expressing cells [Figures 5B,C, One-Way ANOVA, *F*_(2, 11)_ = 227.30, *p* < 0.001; *post-hoc* bilateral Student's *t*-test *p* < 0.001], whereas the volume of the granule cell layer did not change between groups [one-way ANOVA, *F*_(2, 11)_ = 1.51, *p* = 0.27]. In order to test whether the D-serine treatment also increased the proliferation of neuroblasts, we immunostained for DCX and Ki-67 and measured the percentage of double labeled cells. Compared to NaCl, D-serine treatment showed no significant difference in the proportion of DCX-expressing cells which also expressed Ki-67 (NaCl 5.72% ± 0.85 vs. D-serine 4.36% ± 0.73, Student's *t*-test *p* = 0.33, data not shown). Thus, D-serine increased the number of immature neurons but not the proliferation of neuroblasts. The number of BrdU-labeled cells was significantly increased in D-serine injected animals [Figures 5D,E, One-Way ANOVA, *F*_(2, 11)_ = 10.73, *p* < 0.01; *post-hoc* bilateral Student's *t*-test *p* < 0.01], indicating an effect of D-serine on the survival of newborn cells. Finally, we examined differentiation of new cells into neuronal lineage by measuring the proportion of BrdU-labeled cells that also expressed Neu-N (Figures 5F,G). In control and NaCl mice, neurons accounted respectively for 86 ± 2% and 85 ± 1.7% of the surviving BrdU-positive cells as compared to 91 ± 1% in D-serine-treated mice [One-Way ANOVA, *F*_(2, 8)_ = 1.8, *p* = 0.24], indicating that D-serine did not significantly increase neuronal differentiation. When the number of surviving cells was multiplied by the fraction of cells that differentiated into neurons, we found that D-serine-treated mice had 39.8 ± 1.1% more newly-formed neurons than non-treated mice [Figure 5F, One-Way ANOVA *F*_(2, 11)_ = 16.67, *p* < 0.001; *post-hoc* bilateral Student's *t*-test *p* < 0.001]. Thus, D-serine treatment during the fourth week after cell division increased the survival of new neurons, but did not modify their differentiation.

## Discussion

In this study, we tested the effect of repeated injections of D-serine on hippocampal adult neurogenesis. We found that *in vivo*, 8 days of D-serine administration increased cell proliferation as well as the number of both RGL cells (type-1) and TAP cells (type-2) and slightly increased the proliferation of RGL cells. D-serine applied to adult hippocampal neural progenitors in culture also increased cell number suggesting a direct effect of D-serine on adult NSCs. Finally, when administered during the critical phase for activity-dependent survival, D-serine increased the survival of newborn neurons. Since this critical phase lasts until the end of the first month after cell division (Kempermann et al., 2003), the surviving neurons observed after D-serine treatment are expected to survive throughout the entire life of the animals. Thus, D-serine increased adult neurogenesis by acting on several steps of this process and may result in long-lasting changes in the granule cell layer.

Together, these results are relevant to the effect of D-serine on learning and memory. Indeed, newborn neurons in the hippocampus are involved in hippocampal dependent learning and memory (Dupret et al., 2007; Ming and Song, 2011; Gu et al., 2012). The performances of animals in hippocampal-learning tasks are highly correlated with the rate of adult neurogenesis in the hippocampus. For example, voluntary running strongly increases the proliferation of adult neural stem cells in the dentate gyrus and newborn neurons survival (Van Praag et al., 1999b; Snyder et al., 2009) and improves the performance of animals in a water maze (Van Praag et al., 1999a). Inversely, ablation studies lead to decreased performances in hippocampal-dependent learning (Saxe et al., 2006; Dupret et al., 2007; Imayoshi et al., 2008; Massa et al., 2011; Lemaire et al., 2012) and more recently, the optogenetic inactivation or stimulation of new neurons induced memory deficits or improvements, respectively (Alonso et al., 2012; Gu et al., 2012). Thus, by increasing neurogenesis, D-serine may improve learning performances. Further behavioral experiments combined with D-serine treatment and ablation of neurogenesis will enable to test the role of adult neurogenesis in the D-serine-mediated learning improvements.

The effect of D-serine on neuronal survival is consistent with the role of NMDA receptor activation in the survival of adult-born neurone: Neurons generated during adulthood undergo a critical time-window for their survival during the third week after cell division, during which the cell-specific knockout of the NR1 subunit in adult-born neurons dramatically decreases their survival (Tashiro et al., 2006). Inversely, increased activity correlates with increased survival (Kempermann et al., 1998), an effect that may be due to glutamate, since this neurotransmitter enhances neuronal survival (Platel et al., 2010; Kelsch et al., 2012) and D-aspartate, a NMDA receptor agonist produced by newborn neurons, induces the dendritic maturation and survival of these cells (Kim et al., 2010). Thus, the effect of D-serine on new neurons survival may be mediated by an increase in NMDA receptors activity on these cells.

In contrast, the mechanism of action of D-serine on cell proliferation remains less clear and could be mediated by an indirect effect of D-serine on hippocampal network activity or by a direct effect on neural stem/progenitor cells, or both. In favor of the former possibility, adult NSCs proliferation and newborn neurons survival are tightly regulated by hippocampal network activity: High frequency stimulations of the perforant path increase NSCs proliferation and newborn neurons survival (Bruel-Jungerman et al., 2006; Chun et al., 2006; Stone et al., 2011). Moreover, mice placed in an enriched environment showed an increase of both proliferation and survival of newborn neurons in the DG (Kempermann et al., 1997; Van Praag et al., 2000; Tashiro et al., 2007). Inversely, a decrease in neuronal activity decreases neurogenesis (Li et al., 2009; Sun et al., 2009; Krzisch et al., 2013). Thus, by increasing NMDA-dependent neuronal activity (Wake et al., 2001; Xie et al., 2005), D-serine treatment could result in increased cell proliferation. Alternatively, in favor of the hypothesis of a direct action of D-serine on the NMDA receptors of stem/progenitor cells, studies using electrophysiological recordings have shown that RGL cells express NMDA receptors (Wang et al., 2005; Nacher et al., 2007) and our experiments showed that D-serine increased RGL cells proliferation *in vivo* and NPCs proliferation *in vitro*. However, previous studies have shown that, in the SGZ, NSC proliferation is strongly decreased by NMDAR activation (Cameron et al., 1995). Furthermore, a recent study (Huang et al., 2012) showed that D-serine treatment did not change the proliferation of early progenitor cells *in vitro*, although the discrepancy between this earlier study and our observations may arise from the treatment duration (48 h vs. 8 days, respectively) or the origin of the cells (mouse vs. rat). Thus, the mode of action of D-serine remains unclear and the insight gained *in vitro* studies for the understanding of the regulation of the neurogenic niche remains limited and, further *in vivo* experiments using cell autonomous approaches will be necessary to determine the origin and the targets of D-serine in the neurogenic niche.

Together, our results indicate that D-serine administration increases the proliferation of stem/progenitor cells and the survival of new neurons. It remains, however, unclear whether *in vivo*, D-serine release plays a role in the regulation of adult neurogenesis. (Huang et al., 2012) have shown that stem/progenitor cells in the subventricular zone release D-serine and that a blockade of D-serine synthesis reduces cell proliferation *in vitro*, suggesting an autocrine regulation mechanism. However, D-serine has been reported to be secreted by other cells, including astrocytes and neurons (Radzishevsky et al., 2013). Of particular interest, the release of D-serine from astrocytes is triggered by the activation of astrocytic AMPA/kainate receptors (Schell et al., 1995). In the hippocampus, astrocytic territories span tens of micrometers (Bushong et al., 2002) and can extend from the hilus or the molecular layer to the subgranular zone of the dentate gyrus. These cells are therefore ideally located to relay signaling between synaptic activity and the neurogenic niche. Although still speculative, the controlled release of D-serine by astrocytes in the neurogenic niche may be a mechanism coupling neuronal activity in specific territories of the dentate gyrus with the local proliferation of stem/progenitor cells. Future experiments aimed at interfering with astrocytic D-serine synthesis or release may shed light on the regulation of endogenous D-serine in the adult hippocampus and on the processes regulating adult neurogenesis. These mechanisms are relevant to our understanding of the regulation of adult neurogenesis and its use as a target for cognitive impairment.

## Author contributions

Conceived and designed the experiments: Sebastien Sultan, Elias G. Gebara and Nicolas Toni. Performed the experiments: Elias G. Gebara, Sebastien Sultan and Kristell Moullec. Analyzed the data: Sebastien Sultan and Elias G. Gebara. Wrote the paper: Sebastien Sultan, Elias G. Gebara and Nicolas Toni.

### Conflict of interest statement

The authors declare that the research was conducted in the absence of any commercial or financial relationships that could be construed as a potential conflict of interest.
